# Comment on "Does the presence of radiculopathy affect sleep quality and lower extremity functionality in neuropathic low back pain?"

**DOI:** 10.1590/1806-9282.20240711

**Published:** 2024-09-02

**Authors:** Bilgehan Kolutek Ay, Mustafa Tuna

**Affiliations:** 1 Kahramanmaraş University, Faculty of Medicine, Department of Physical Medicine and Rehabilitation – Kahramanmaraş, Türkiye.; 2 Şanlıurfa Training and Research Hospital, Department of Physical Medicine and Rehabilitation – Şanlıurfa, Türkiye.

Dear Editor,

First and foremost, we would like to express our gratitude to our esteemed authors for their interest in and contribution to our article^1^. This study was conducted with 79 patients diagnosed with lumbar disc herniation (LDH) presenting neuropathic-type low back pain^2^. Patients included in the study were those who sought treatment at the clinic due to chronic low back pain. Rather than the variety of current treatments received, whether patients were taking medications that could cause neuropathy or disrupt sleep quality was more important for this study. Patients diagnosed with sleep disorders or neuropathy were not included in the study, as stated in the exclusion criteria.

The exclusion criteria of this study included past disc surgery, vasculitis, spinal cord injury, and conditions such as HSV and HIV. Patients with inflammatory, infectious, congenital, or traumatic pathologies involving the lumbar region were not included in the study. Spinal stenosis can occur due to pressure from a herniated disc inherently^3^; hence, congenital or structurally caused spinal stenosis could have been added to the exclusion criteria. Although the inclusion criterion of having received a diagnosis of LDH partially explains this situation, the condition of spinal stenosis could have been elaborated further.

The role of depression and anxiety disorders in both chronic pain and sleep disorders is well-established. This issue is discussed in the discussion section of our article. Due to the numerous studies in the literature on this topic, the potential contributions of our findings were limited in terms of positivity or negativity. The main aim of this study was to investigate the impact of electroneuromyographically (ENMG) proven radiculopathy on sleep and lower extremity functions and compare it with patients without radiculopathy.

During routine examinations of our patients, their habits are questioned. None of the patients included in this study had alcohol or stimulant substance dependencies, although some participants were smokers. This information could be mentioned as general knowledge and evaluated in the table assessing sleep quality.

Conditions such as weakness accompanying neuropathic pain (NP), allodynia, and decreased deep tendon reflexes mentioned in the introduction of our article are not prerequisites for the diagnosis of NP. When the referenced article is reviewed, it is observed that NP can occur without these findings^4^. Given our clinical experience encountering NP exactly as described by the authors^1^ and our belief that the concept of NP needs to be evaluated more broadly, this study was conducted. NP can persist in individuals with no neurological deficits, no disc herniation, and non-specific lower back pain, either related to disc herniation or due to untreated prolonged nociceptive pain and pathologies in pain processing pathways^5^. This issue is discussed in the discussion section of our article. Furthermore, upon reviewing the inclusion criteria, it was observed that 148 participants who underwent ENMG and were diagnosed with disc herniation were examined. There were also patients with ENMG findings who did not meet the DN4 criteria for NP, and these patients were not included in the study.

Contrary to what the authors mentioned, we did not have an evaluation or conclusion similar to patients with and without radicular pain in this study. When the inclusion criteria were examined, all patients had radicular pain. Our differentiation was based on whether patients had radiculopathy, according to ENMG findings.

The concept of chronic pain and neuropathic pain is a condition with many causes, and research into its etiopathogenesis continues. In this sense, every study will contribute to shedding light on the subject.
